# The promotion of active aging through older adult education in the context of population aging

**DOI:** 10.3389/fpubh.2022.998710

**Published:** 2022-10-10

**Authors:** Kexin Zhang, Chengxia Kan, Youhong Luo, Hongwei Song, Zhenghui Tian, Wenli Ding, Linfei Xu, Fang Han, Ningning Hou

**Affiliations:** ^1^Department of Endocrinology and Metabolism, Affiliated Hospital of Weifang Medical University, Weifang, China; ^2^Clinical Research Center, Affiliated Hospital of Weifang Medical University, Weifang, China; ^3^Department of Pathology, Affiliated Hospital of Weifang Medical University, Weifang, China

**Keywords:** active aging, education, older adult education, population aging, public health

## Abstract

We have entered an era of population aging, and many public health problems associated with aging are becoming more serious. Older adults have earlier onset of chronic diseases and suffer more disability. Therefore, it is extremely important to promote active aging and enhance health literacy. These involves full consideration of the need for education and the provision of solutions to problems associated with aging. The development of OAE is an important measure for implementing the strategy of active aging, and curriculum construction is a fundamental component of achieving OAE. Various subjective and objective factors have limited the development of OAE. To overcome these difficulties and ensure both active and healthy aging, the requirements for active aging should be implemented, the limitations of current OAE should be addressed, system integration should be increased, and the curriculum system should be improved. These approaches will help to achieve the goal of active aging. This paper discusses OAE from the perspective of active aging, based on the promotion of health literacy and provides suggestions to protect physical and mental health among older adults, while promoting their social participation. The provision of various social guarantees for normal life in older adults is a new educational concept.

## Introduction

The world's population is aging rapidly, and older adults are expected to live longer (Table 1) (1–3). Over the last few decades, population trends indicate earlier onset of many chronic diseases and an increased incidence of multimorbidity in older adults (4, 5). As these problems emerge, older adults may experience more disability and worse quality of life (6). Discussions of population aging and its effects have become an important and popular topic. In the context of population aging, the role of older adult education (OAE) has become increasingly prominent. Although there have been many studies of OAE, there is minimal available information concerning OAE with a focus on active aging. In this review, we analyze the current status of OAE in the context of active aging and propose measures for reforming OAE. We also aim to increase investment in OAE, ensure greater focus on older adults, and promote active aging.

**Table 1:** Composition of the world and regions by age (thousands).

**Age (ys)**	**World**	**Sub-Saharan Africa**	**Northern Africa and Western Asia**	**Central and Southern Asia**	**Eastern and South-Eastern Asia**	**Latin America** **and the** **Caribbean**	**Oceania (excluding Australia and New Zealand)**	**Australia/New Zealand**	**Europe and Northern** **America**
0–4	671,477	178,145	57,283	186,182	139,117	49,740	1,585	1,848	57,578
5–9	683,612	158,822	57,761	190,450	157,682	52,115	1,527	1,945	63,309
10–14	659,934	141,832	52,568	191,437	153,468	52,702	1,433	1,939	64,556
0–14	2015,023	478.798	167,612	568,068	450,267	154,557	4,546	5,732	185,443
15–19	623,561	122,009	46,490	191,205	144,918	53,222	1,323	1,821	62,574
20–24	600,697	102,883	44,040	184,496	148,161	53,930	1,212	2,024	63,951
25–29	593,832	87,830	43,869	175,430	160,818	53,124	1,119	2,307	69,335
30–34	606,213	75,538	43,237	165,661	189,230	51,521	1,019	2,341	77,666
35–39	558,995	63,840	40,898	150,661	173,451	48,958	895	2.221	78,069
40–44	500,404	51,940	36,308	130,567	157,458	45,066	773	1,976	76,317
45–49	476,003	41,136	30,604	114,486	171,997	40,551	663	1.975	74,590
50–54	450.467	33,356	25,717	99,013	177,995	36,433	563	1.938	75,453
55–59	400,890	26,176	20,978	84,515	158,135	32,616	464	1.859	76,146
60–64	321,938	19,770	16,084	69,950	113,630	27,130	341	1,746	73,289
65–69	276,129	14,449	12,042	53,981	108,558	21,360	227	1,519	63,993
70–74	201,868	9,797	8,138	35,913	77,748	15,655	146	1,339	53,131
75–79	128,623	5,949	4,882	21,543	48,670	10,602	85	967	35,924
80–84	86,296	2,942	2,867	12,349	32,225	6,625	43	650	28,595
85–89	45,540	1,121	1,257	5,413	17,708	3,239	17	384	16,401
90–94	17,902	327	374	1,715	6,675	1,176	5	190	7,440
95–99	4,322	67	66	341	1,613	291	1	55	1,887
100+	593	11	7	42	234	43	0	7	249

## Current status of active aging

**Figure 1:**
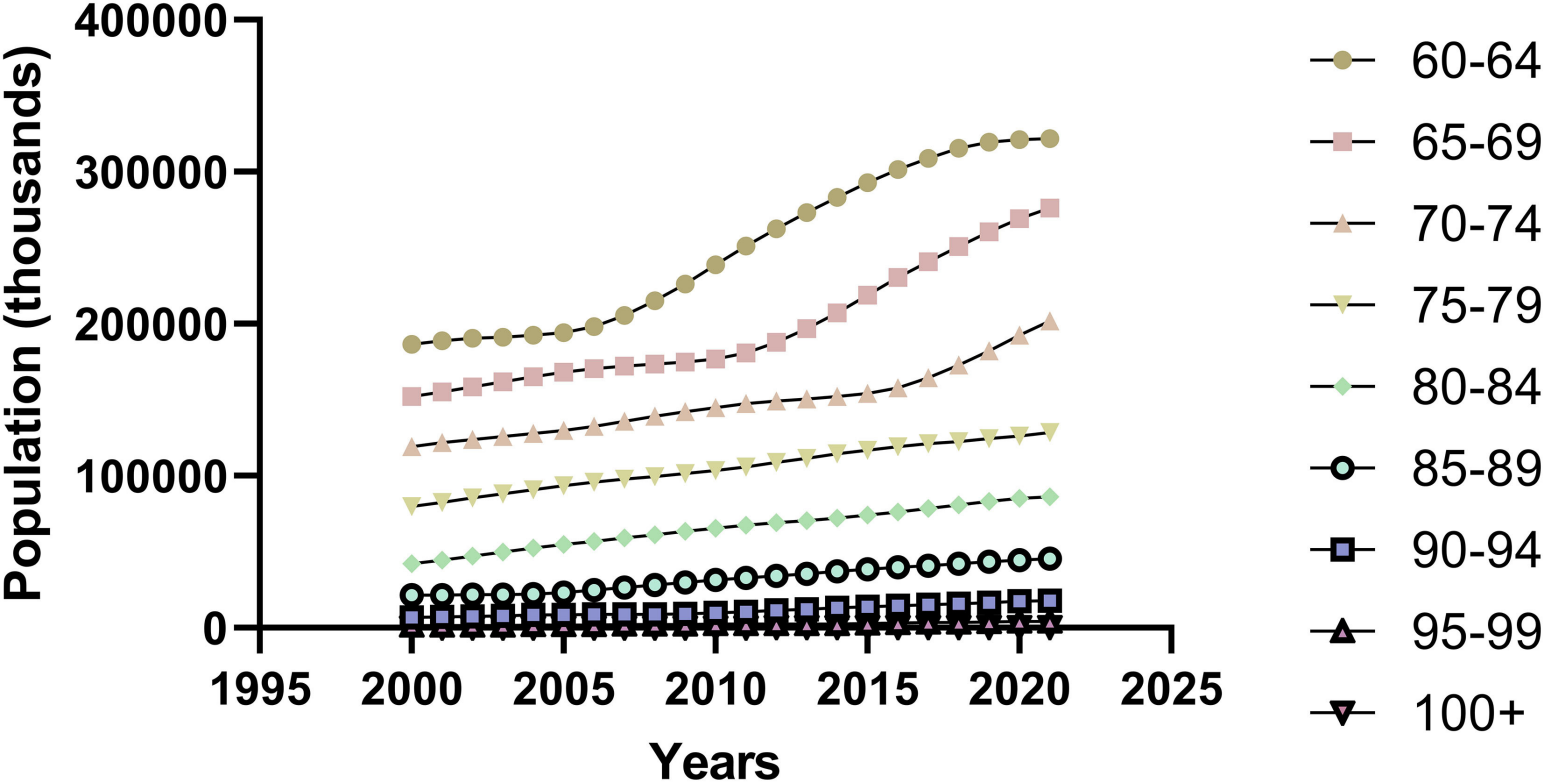
Trends in the elderly population aged 60 years or older from 2000 to 2021.

**Figure 2:**
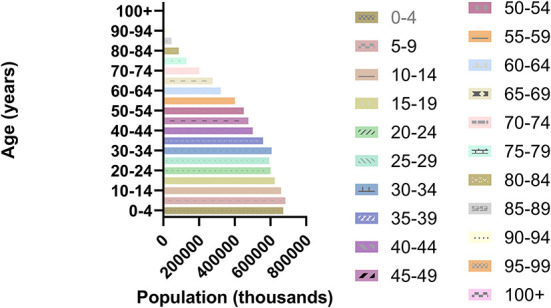
World population composition by age group in 2021.

**Figure 3:**
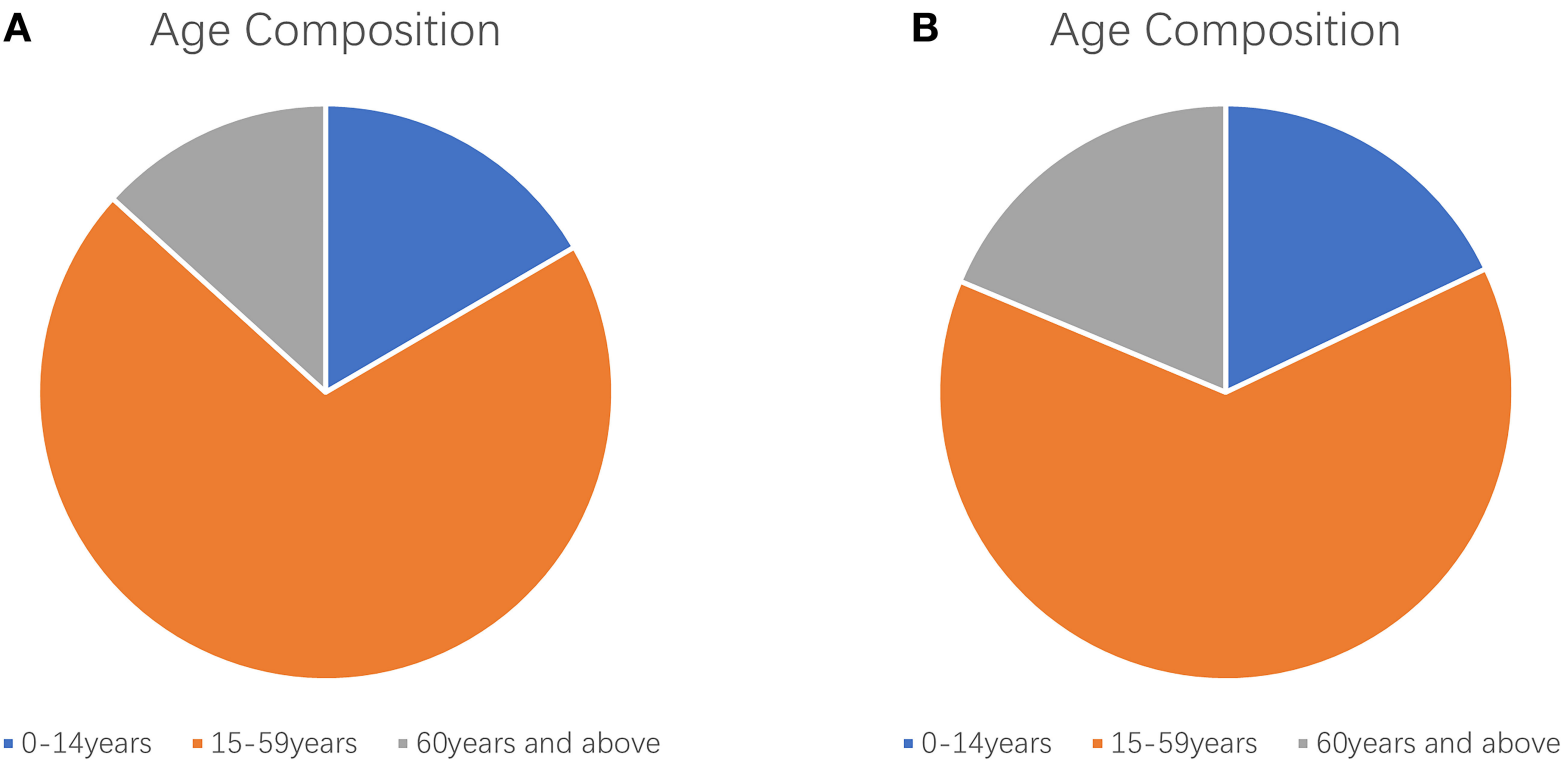
Age composition of the Chinese census. **(A)** Age composition of the sixth national census of China; **(B)** Age composition of the seventh national census of China.

We define aging (also known as senescence) as the intrinsic physiological decline that occurs as an organism ages, leading to decreased fertility and reproduction, as well as increased risks of age-related morbidity and mortality (7). In the past 20 years, the world's older population has increased (Figures 1, 2). For example, the Seventh National Census of China had a greater proportion of people aged ≥60 years, compared with the Sixth National Census (Figure 3); the proportion of people aged ≥60 years increased by 5.44% (Data from http://www.stats.gov.cn/).

In this context, the concepts of “successful aging” (8, 9), “healthy aging” (10, 11), and “active aging” (12, 13) have been introduced to reflect positive responses to aging in various countries worldwide. The concept of successful aging was introduced as early as the 1960s. Its connotations include the need for older adults to maintain mental health and normal cognitive function, be socially active, have good interpersonal relationships, and be physically healthy (8, 14, 15). Healthy aging was introduced by the World Health Organization; its core concepts include physical health, mental health, and good social adjustment. Active aging is another level of healthy aging (10). Notably, active aging was defined in 2002 by the World Health Organization as the process of gaining access to the greatest possible variety of opportunities for health, participation, and security, with the goal of improving the quality of life in older adults (16–19). The concept of active aging includes the concepts of “healthy aging” (11), “successful aging” (9), and “productive aging” (20); it also has a more systematic and comprehensive core meaning. Over the past two decades, the concept of active aging has been applied to social, scientific, psychological, and medical disciplines. Active aging is regarded by international political groups and researchers as an important component of efforts to address the challenges of population aging (21). Active aging now highlights the need for a population aging approach that involves physical, social, and mental health, while encouraging social participation and providing adequate protection, security, and care based on the needs of older adults (22). Thus, active aging is an effective response to the experience of aging. An important challenge for the current aging society comprises the optimization of active aging to enhance survival and development among older adults, promote their social participation and overall development, and improve their standard of living.

## Development of OAE

### Nature and content of OAE

Population aging is a global phenomenon that requires action to promote well-being and prevent disease (23). OAE is an informal component of adult education and the final stage of lifelong education (24). With the increasing problem of aging, the retired older population is also increasing and has become a social group that cannot be ignored. The OAE in this article is education for retired seniors 60 years and older and those who are not working. The development of OAE is essential for developing the careers of older adults and improving their participation; it is also an important initiative for actively managing population aging, modernizing education, and promoting lifelong learning.

OAE has a long history of development; its definition and content vary according to the physical and mental characteristics, needs, and preferences of older adults. In the past, the range of courses offered in OAE was limited and focused only on the interests of older adults, such as calligraphy, painting, instrumental music and dance (24). Recently, because of the emphasis on active aging, the curriculum has focused on the physical and mental health of older adults and included some new skills knowledge (e.g., computer networks, mobile phones, and computer use) (25). These changes continue to enrich OAE. This review of OAE focused on studies of older adults entering retirement; it was written with the goal of improving their physical and mental health, promoting their social participation, and providing them with various safeguards for normal life through the delivery of OAE.

### The purpose of developing OAE

OAE emerged as a new type of education in the 1970s, and it developed as one of the positive responses to the aging of the world (26). OAE is for seniors 60 years and older who are retired or have plenty of leisure time. OAE can enrich the lives of older adults and enhance their quality of life. Moreover, OAE can fully use the older adults' strengths and create a “society of learners” by influencing the people around them with practical actions. The essential features of the “society of learners” are universal and lifelong learning. “Society of learners” is a basic form of future society. And senior education, as an essential part of the last stage of lifelong education, is of great significance in building and improving a harmonious and continuously developing “society of learners”. Finally and most importantly, OAE can help older adults improve their health literacy and achieve prevention and monitoring of diseases.

## Challenges of OAE

Currently, there are several problems with OAE that hinder the achievement of active aging. The lack of educational opportunities and resources partially limits the development of OAE. Additionally, low motivation to participate in education exacerbates the problems in the development of OAE; this prevents it from rapidly achieving better integration into the new contemporary environment. Finally, limitations of the educational model and a lack of professional staff also hinder the development of OAE. There is a need for efforts to address the current problems in the development of OAE and actively promote educational exploration through the optimization of existing educational strategies. The specific limitations of OAE in the context of active aging are as follows (Table 2).

**Table 2:** Problems and solutions for older adult education.

**Problems of older adult education**	**Solution measures**
Insufficient popularization and lack of social participation awareness	Full construction and promotion of OAE University (1) Develop and improve the OAE University (2) Broaden publicity channels and organize OAE talks to raise awareness of older adults' participation and promote OAE
Deficiencies of the teaching mode	Diversification of teaching modes (1) Improve face-to-face courses (2) Provide online teaching support (3) Explore a mix of online and offline teaching models (4) Develop mutual learning
Lack of high-quality courses	Develop high-quality courses (1) Require the involvement of the public health system (2) Provide health literacy courses (3) Conduct regular health assessments (4) Develop and improve a systematic mental health education curriculum

### Insufficient popularization and lack of social participation awareness

There is a serious imbalance throughout the OAE developmental process (27).

Few countries have focused on developing OAE universities, but the establishment and development of OAE in China are clearer. OAE universities were established first in 1983. By the end of 2019, there were more than 62,000 OAE universities with more than 8 million students in China. The OAE universities in China refer specifically to universities, colleges, schools and institutions that conduct OAE and are organized or supported by the government, enterprises or social organizations. There were more than 250 million people over the age of 60 in China, and only 5% of them were older students, indicating that there was a big gap between the existing scale of OAE education and the participation rate of older people in education under active aging (28). First, OAE is achieved through the vehicle of the OAE universities (24). OAE universities are places for retired seniors to relax, have fun, study and make friends, mainly to promote senior health; they focus on the learning, well-being, and quality of life of older adults to ensure their healthy development. The OAE universities that currently exist are insufficient for the large population of older adults. Second, OAE in the context of active aging has been discussed at the academic level by many experts and researchers, and it has been implemented in some regions; however, it has not been sufficiently promoted in terms of publicity (29). Furthermore, older adults, as the recipients of OAE, are not fully aware of the concept of “lifelong learning” and the importance of participating in OAE; they do not realize the vital role of OAE in improving health literacy, relieving anxiety, and benefiting their own development (particularly their social development). Finally, despite the global digitalization process, older adults have a limited acceptance of information and network technologies, applications, and innovation capabilities; the existence of a “digital divide” hinders active aging (30).

### Deficiencies in teaching models

The most common and effective teaching model is face-to-face interaction with accessible communication and timely communication. This model is also applicable to OAE. However, the limited audiences in this model reduce its usefulness (31). The current coronavirus disease 2019 pandemic is an unprecedented situation that has affected all aspects of society, including education; the implementation of face-to-face courses has also been profoundly affected (31, 32). Complete reliance on face-to-face instruction for OAE will affect the speed of teaching and learning, as well as the participation and motivation of learners. Thus, when establishing the teaching model for OAE, there is a need to consider learning ability, physical fitness, and basic health in older adults (24). Educational efforts should focus on maintaining effectiveness while avoiding negative effects on quality of life and health among older adults. However, because of the unique nature of OAE, the knowledge base and comprehension of each older adult learner varies considerably (33); traditional teaching methods cannot be applied to OAE. Currently, most regional OAE institutions use the university education model, without considering the life and educational statuses of older adults; this reduces the effectiveness of OAE and does not support long-term implementation of OAE.

### Lack of high-quality courses

The lack of quality courses for older adult learners continues to be a major barrier to the growth of OAE (24). There is a lack of uniformity and standardization in the curriculum setting of OAE universities, and there are problems such as scarce quantity, scattered topics and uneven quality of courses. The textbooks generally lack the characteristics of OAE education, and they are presented in a single form, with little practicality and low utilization. In China, the curriculum structure is aging, textbooks do not appropriately reflect the educational characteristics of older adults, and > 56% of students believe that textbooks should be more advanced and practical (34). Other countries have similar problems. In Korea, OAE is not limited to general courses; there have been suggestions of additional courses concerning the social welfare system, health and healthcare, civics, and the economy (35). In Peru, the existing curriculum does not meet the real needs of students from a wide range of educational backgrounds (33); few universities have academic educational programs for older adults (33).

The content of the current OAE courses does not focus exclusively on recipients themselves. There is considerable scope to develop OAE courses on health literacy (24). The world's population is aging rapidly, multimorbidity is prevalent among older adults, and older adults are prone to psychological problems that can lead to depression after retirement owing to changes in social status. Therefore, OAE should focus on improving health literacy and protecting the mental health and physical well-being of older adults. In this context, health literacy comprises the ability to find, understand, use, and evaluate health-related information; such an ability is a critical determinant of health (36, 37). As an example of the need for improved health literacy, in December 2009, the Chinese Ministry of Health published the results of the first official health literacy survey involving Chinese residents; only 3.81% of adults aged 65–69 years had adequate health literacy, which was the lowest level among all surveyed age groups (38). In the context of population aging, chronic non-communicable diseases have created a heavy disease burden worldwide; there is increasing multimorbidity, which constitutes a serious threat to safety and quality of life among older adults (38). Additionally, there is a need to monitor psychological disorders; the fast-paced nature of modern life makes it difficult for younger adults to closely observe psychological changes in older adults. Some studies have shown that anxiety disorders are more common in later life; there is some evidence that anxiety disorders are associated with lower levels of education (39). Grossman's theory of health production suggests that health is influenced by healthcare, income level, lifestyle, education level, and living environment (40). There is a close link between an individual's education level and their health (41, 42). Improvements in education level are needed to promote health in older adults (43). Health-related courses should be included to create a high-quality curriculum.

### Recommendations for promoting OAE

Active aging represents a major strategic initiative to address aging-related problems. OAE provides reliable conditions for implementing the active aging strategy and a strong incentive to promote active aging. The attainment of a healthy aging society requires efforts to prepare for the challenges and problems associated with rapid population aging (44). In the following three sections, recommendations are provided for improvements to OAE (Table 2).

### Full construction and promotion of OAE university

OAE universities are intended to maintain physical autonomy and independence in older adults by providing learning opportunities, recreation, and social activities that can enhance their quality of life (45). OAE universities serve as the main avenues for educating older adults. The development and improvement of OAE can support healthy aging (46). First, more educated older adults have higher health literacy, so there is a positive correlation between OAE and health literacy; such education is associated with better health and survival (37). Second, higher levels of education help individuals to build psychosocial resources; increased education can prevent cognitive impairment in older adults (47). Third, people with higher levels of education are more likely to adopt positive health behaviors, compared with people who have lower levels of education; in this context, positive health behaviors include regular exercise, moderate alcohol consumption, and avoidance of smoking. All three of these behaviors are associated with better health and a lower mortality rate (48).

Education can help older adults maintain their independence, preserve their dignity, and have a positive attitude toward aging. It is important to promote active aging through education (49, 50). However, the current lack of outreach capacity and insufficient supply of OAE are problems that continue to restrict the development of OAE. To increase the motivation of older adults to participate in OAE (40), countries should formulate a series of support strategies for developing OAE according to national conditions; such strategies will enable OAE to be fully valued by older adults. The promotion of OAE involves broadening channels, increase government investment in OAE at all levels, introduce social capital, and use internet resources and television news for publicity. Additionally, OAE services should be directly accessible through the community. Communities and senior activity centers can hold offline experience courses and lectures to raise awareness of OAE among older adults.

### Diversification of teaching models

Unlike other forms of education, OAE has flexibility in terms of educational content and form, as well as diverse educational objectives and unique information; these characteristics determine the diversity of OAE models (24). First, face-to-face courses can be improved; for example, teaching sites can be established in homes or community organizations to facilitate participation by older adults who live in nearby communities and have limited mobility. In Japan, small and community-based learning opportunities have been popular; they remain active (25). Second, online teaching support can be provided for OAE. Online teaching is convenient and fast, not limited by time and space, and requires only a mobile phone or a computer. The development of online courses can offset the limitations of traditional forms of education (51). Additionally, a mixed online and offline teaching model is the focus of active exploration (52); joint instruction that involves OAE universities and local colleges can be developed to introduce quality courses of interest to older adults, while enhancing the delivery of educational content. Finally, mutual learning can be promoted, which involves a shift in learner and teacher roles (25). Members of OAE universities could invite members of other classes to discuss what they have learned in past courses (25).

### Development of high-quality courses

High-quality curriculum is an essential component of OAE, which determines the quality of education (24). The first step in creating a high-quality curriculum involves the improvement of content for older adults. Because health maintenance is a critical consideration for older adults, most OAE courses should include concepts that help to promote health in their learners (25). First, this focus requires the public health system to provide programs that invest in the long-term prevention of chronic diseases and promote health recovery for all learners in OAE universities (53). Second, health literacy science and mental health education courses should be offered because a positive mindset and a good lifestyle are essential for active aging (54). These courses should include lectures to disseminate specific knowledge concerning the prevention of chronic diseases and cancer (i.e., basics of disease prevention), encouragement of healthy lifestyle maintenance with a sensible diet, moderate exercise, smoking cessation, alcohol restriction, and psychological balance; and promotion of healthy aging through appropriate physical activity classes (55). Online classes should be conducted to improve medication-related knowledge in older adults, which can help them to use medications more safely (56). Furthermore, health behavior assessments should regularly be conducted at OAE universities; these will enable early detection, treatment, and management of diseases. Health behavior assessments include evaluation of smoking status, alcohol consumption, and physical activity, as well as health screening and body mass index calculation; together with dietary habits, these components are regarded as basic health behaviors (38). Based on these evidences, we suggest that OAE universities should develop a systematic psychological education curriculum for older adults. They should also contact mental health education experts or authoritative psychological counselors to deliver mental health knowledge through lectures. OAE universities should arrange professional assessments of mental health in older adults. When appropriate, efforts should be made to communicate with the family members of older adults (by phone or in person) to ensure that younger adults understand the psychological changes in their older family members. Older adults should receive warm and caring support; professional counselors should be available for early intervention and treatment when serious problems are identified.

## Conclusion

Population aging is a growing problem; active and effective management of this problem is urgently needed. The development of OAE is a long-term process; it is inevitable that various bottlenecks will occur during the development process, which requires systematic coordination. Governments and authorities at all levels should create conditions for participation, while actively regulating and guiding the development of OAE. They should also expand curriculum reform, strive to improve educational quality, and make full use of existing educational resources to effectively alleviate the contradiction between supply and demand in OAE. Professionals should carefully monitor the physical and mental health of older adults; families should provide as much companionship and care as possible; and older adults themselves should improve their health literacy and actively participate in the learning process. The investment of robust effort to support older adults, particularly with respect to OAE, is an important aspect of promoting balanced development of society overall.

## Author contributions

FH and NH: conceptualization and writing—review and editing. KZ and CK: methodology, software, visualization, and writing—original draft. YL, HS, ZT, WD, and LX: methodology and writing—review. All authors contributed to the article and approved the submitted version.

## Funding

This study was supported by grants from National Natural Science Foundation of China (81870593 and 82170865), Natural Science Foundation of Shandong Province (ZR2020MH106), Shandong Province Higher Educational Science and Technology Program for Youth Innovation (2020KJL004), and Yuandu scholars (2021).

## Conflict of interest

The authors declare that the research was conducted in the absence of any commercial or financial relationships that could be construed as a potential conflict of interest.

## Publisher's note

All claims expressed in this article are solely those of the authors and do not necessarily represent those of their affiliated organizations, or those of the publisher, the editors and the reviewers. Any product that may be evaluated in this article, or claim that may be made by its manufacturer, is not guaranteed or endorsed by the publisher.
